# The launch of EMBO reports 25 years ago

**DOI:** 10.1038/s44319-025-00571-w

**Published:** 2025-09-04

**Authors:** Frank Gannon

**Affiliations:** QIMR Berghofer Medical Research Institute, Brisbane, QLD Australia

**Keywords:** Science Policy & Publishing

## Abstract

EMBO’s decision to start a new journal 25 years ago was not an easy one but was made to address changes in the publishing world. When looking at EMBO reports in hindsight, it was indeed a much-needed addition to the range of existing journals.

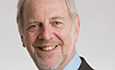

“Should we start a second journal?” “Do we need a second journal?” “Are there too many journals already?” Those were questions that EMBO debated in the late 1990s. The organization already had a publication: *The EMBO Journal*, started in 1982. It was a benchmark for quality and outstanding research, and it generated additional income for the fellowship and workshop programs, and started the Young Investigators program. It was a great success, and because of that, there were concerns that a new journal from EMBO, if it were not of the same stellar standard, could detract from *The EMBO Journal* and EMBO could become associated with doing something that was second best.

On the other hand, the world outside EMBO was not static. Electronic publications were starting in parallel with the print copy. The subscription model was increasingly being challenged by the Open Access movement. The number of researchers in the life sciences was steadily increasing, and so did the number of publications. The latter meant that top journals could place a very high bar on acceptance, which, in turn, required that the authors needed a very significant body of data using different approaches to prove their point. This was beyond the reach of many labs with limited funding and time pressures for graduates and postdocs to advance their careers. The papers in *The EMBO Journal* had been of the highest quality, but the journal addressed the publication needs of a small section of the scientific community.

We noted that *Nature* and *Cell* had started a series of new journals under their brand names. As the EMBO membership covers all aspects of the molecular life sciences, one option would be to use the EMBO network to start a similar series of EMBO journals focused on each discipline, such as structural biology or neurobiology. However, there was a strong feeling that one benefit of the broad scope of disciplines covered by *The EMBO Journal* was that it introduced readers to a wide range of different concepts and results, which would be lost if EMBO launched new journals dedicated to specific areas.

With time, the discussions crystallized around the things that *The EMBO Journal* did *not* do. A clear answer was short papers with a single insight message coming from any area of molecular biology. A journal covering such articles with high standards of peer review would help to get new concepts to the scientific community more rapidly. The intergovernmental organization that supports EMBO had expanded to include a number of new member states, and with it came an awareness of the fiscal constraints under which some of their researchers worked. Attending international meetings could be a rare event for them, hence meeting reports to disseminate the latest research directions was a way to serve their needs. Expert reviews had been part of *The EMBO Journal*, but with the increasing number of articles in that journal, they had become more sporadic.

The most innovative concept of *EMBO reports* was a front section that explored the interplay between science and society. EMBO had a track record of such engagement dating back to the discussions on recombinant DNA technology, where John Tooze, my predecessor as Director of EMBO, played a crucial role. In the late 1990s, those discussions had moved on to Genetically Modified Organisms and the consequences of the first draft of the Human genome. We also noted that a European perspective was largely missing from the two journals that were widely read for such background information: *Science* and *Nature*, which had a more US-centered coverage back then.

Eventually, the decision to start a new journal was taken based on the opportunities and needs we had identified. As a bonus, Nature Publishing Group were willing to publish the journal online as well as in print. The name, “*EMBO reports*,” should suggest brevity, impact, currency and the sharp and attention-gathering sound from an explosion. Word was spread through EMBO’s networks of members, fellows, Young Investigators and at courses and workshops. Editors, including one with the breadth required for the Science & Society section, were recruited, and then we waited and hoped. July 1 2000 was the date for “blast-off”. As it was getting closer, we learned, to my horror, that the binding of the printed version required approximately a hundred pages or else it would disintegrate as it could not be adequately glued. It required some insistence on the primary need for quality to reject some of the papers that were submitted during the nervy period before we went to press. But we got there with ninety pages including two viewpoints (on xenotransplantation and on the ethics of the use of embryonic stem cells), two Analysis articles (on the need for greater European support for plant research and the impact of electronic publishing), two reviews, a meeting report, a literature report and ten scientific articles. I wrote the editorial “A new journal—in more than one way”. Cheekily, that was tagged as “Quite Frankly”, but we changed it after a few issues.

What I had overlooked initially was the need for a monthly editorial. Once I adjusted to this challenge, I enjoyed the opportunity to write about a wide range of topics over the next 100 issues. Walking our dog every morning was a great time to reflect on research life, and flights to meetings in Europe were just about the right length to put together a first draft for editing and criticism. Perhaps the most significant of these editorials—starting in 2000—promoted the idea of a European Research Council, which was established in 2007. Twenty-five years after *EMBO reports* started, the scientific publishing world has changed, as is reflected in the accompanying editorial by Howy Jacobs, who followed me as editor. Still, the decision in 2000 to do something new has been vindicated by every measure. *EMBO reports* moved from countdown to launch and is now orbiting with its own special value in a crowded constellation of journals.

